# Supplementary figures and images for: Virtual lab coats: The effects of verified source information on social media post credibility
Source: PLoS One. 2024 May 29;19(5):e0302323. doi: 10.1371/journal.pone.0302323 (PMC11135712; doi:10.1371/journal.pone.0302323)

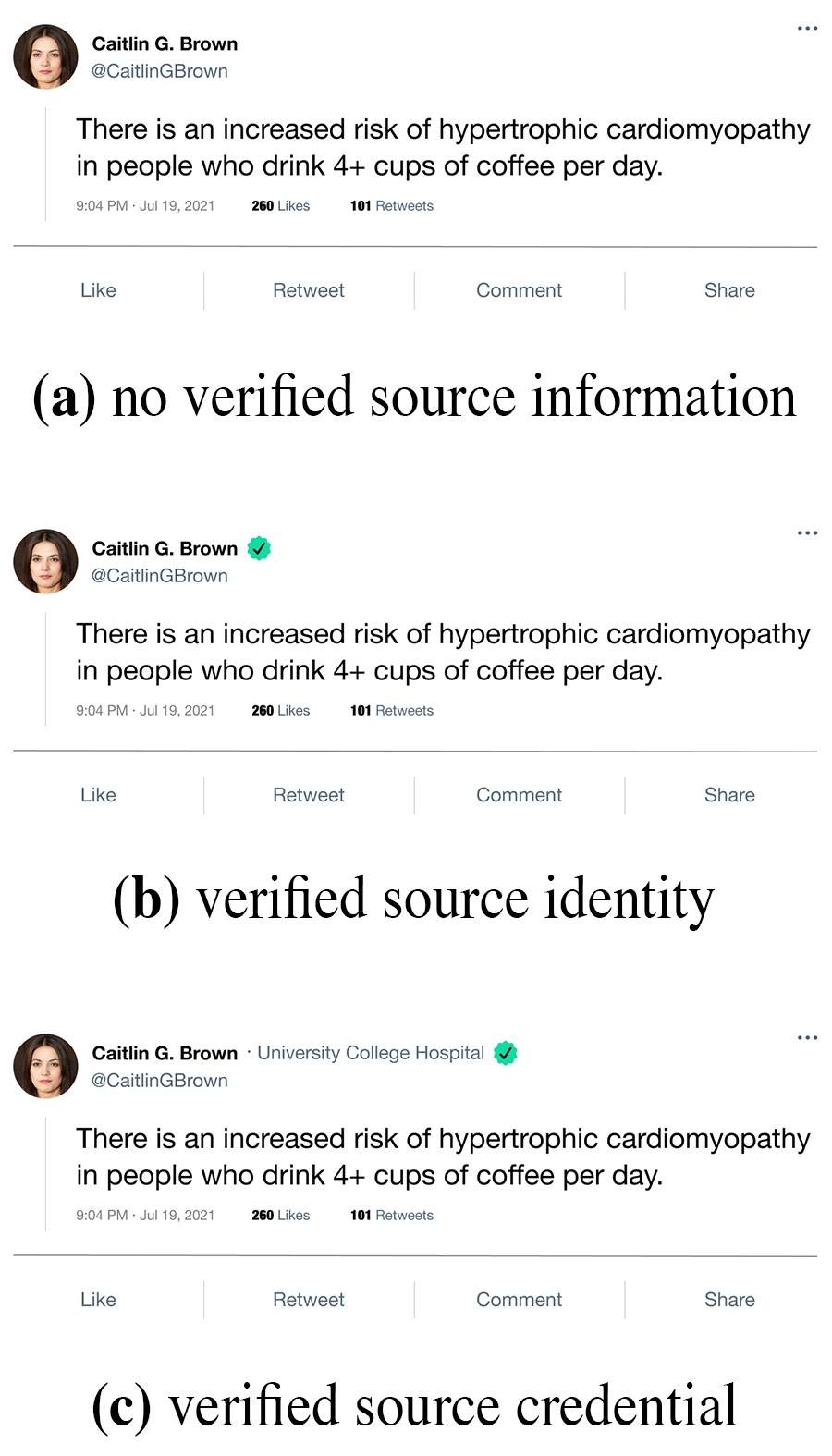

Supplement: S1 Fig — An overview of the stimuli used in the medical context in study 1. Here, the medical signature (c) is considered a relevant attribute, as it is displayed in the medical context. Note that this figure is for illustrative purposes only for two reasons. First, the profile photo is similar but not identical to the one used in the experiment. While the original photo was obtained from Unsplash, this illustrative profile picture was AI-generated through https://thispersondoesnotexist.com. Second, this Figure differs from the original stimulus in that it features a self-designed version of a Tweet for legal reasons. (TIF) [file pone.0302323.s001.tif]

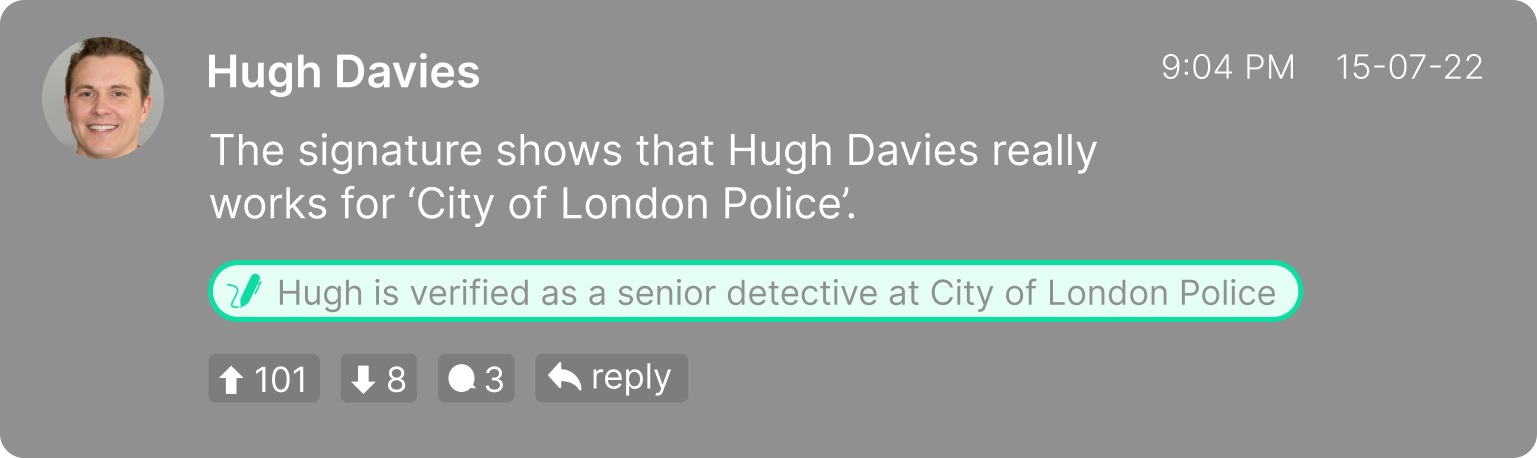

Supplement: S2 Fig — Example social media post including a credential signature. Note that this image was used as support image in explaining the meaning of the various badge and signature designs. Note that, again, this figure is for illustrative purposes only, as the profile photo is similar but not identical to the one used in the experiment. While the original photo was obtained from Unsplash, this illustrative profile picture was AI-generated through https://thispersondoesnotexist.com. (TIF) [file pone.0302323.s002.tif]

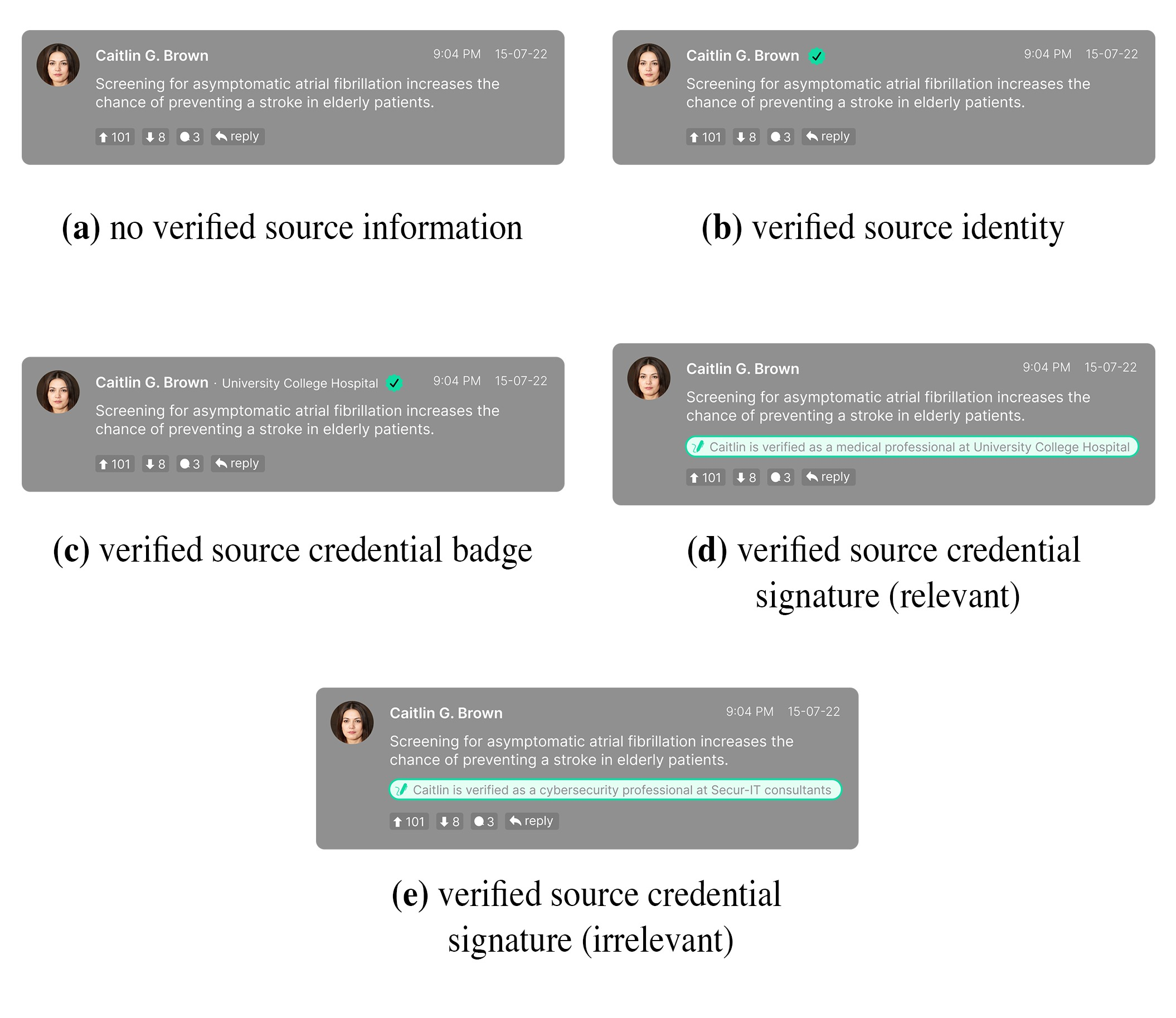

Supplement: S3 Fig — An overview of the stimuli used in the medical context in study 2. Here, the medical signature (d) is considered a relevant credential, whereas the cybersecurity signature (e) is considered irrelevant. Naturally, the opposite holds in case of the cybersecurity context (where only the message contents are replaced). Note that, again, this figure is for illustrative purposes only, as the profile photo is similar but not identical to the one used in the experiment. While the original photo was obtained from Unsplash, this illustrative profile picture was AI-generated through https://thispersondoesnotexist.com. (TIF) [file pone.0302323.s003.tif]
