# Supplementary material for: Virtual lab coats: The effects of verified source information on social media post credibility
Source: PLoS One. 2024 May 29;19(5):e0302323. doi: 10.1371/journal.pone.0302323 (PMC11135712; doi:10.1371/journal.pone.0302323)
Supplement: S1 File — This text was used to explain to the participants how to interpret the different possible badges included in the first experiment. It was explained that a source could have either no badge, an identity-based badge, or a credential-based badge. The text further explained how to interpret the badges, namely as verified identity and verified credential respectively. Lastly, this File differs from the original stimulus in that it features a self-designed version of the Twitter verification icon for legal reasons. (PDF) [file pone.0302323.s004.pdf]

Please read the following information carefully.

Twitter is going to introduce a new type of verification badge.

So far Twitter accounts either didn't have any verification badge or they had a blue verification badge (). A blue verification badge "lets people know that an account of public interest is authentic" (according to Twitter). For example, if the account "The Royal Family" has a blue badge it means that it really is the Twitter account of the Royal Family. We refer to this badge as the "**classical**" badge.

Now, the **new** verification badge lets people verify certain information about them, for example where they work or live. Example: If a person works for the "City of London Police" they could have a verification badge on their Twitter account saying City of London Police.

Hence, Tweets can either have **no**, the **classical** or the **new** verification badge.

Following you will see the same Tweet from before again.
