# Supplementary material for: Virtual lab coats: The effects of verified source information on social media post credibility
Source: PLoS One. 2024 May 29;19(5):e0302323. doi: 10.1371/journal.pone.0302323 (PMC11135712; doi:10.1371/journal.pone.0302323)

Please read the following information carefully.

On some social media platforms, a verification badge lets people know that an account of public interest is authentic. For example, if the account "The Royal Family" has a green badge it means that it really is the social media account of the Royal Family. We call this the “classical badge”.
A social media post with such a **classical badge** looks like this:

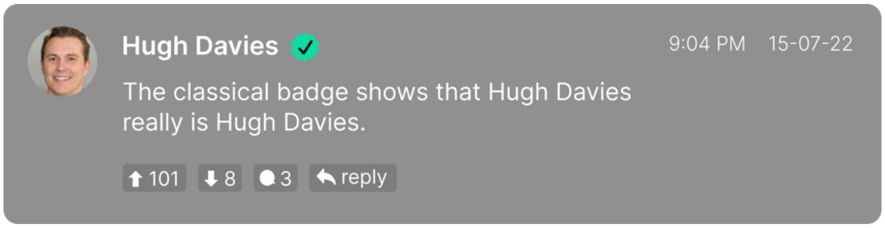

Now, we are experimenting with a new verification badge that lets people verify certain information about them, for example where they work or live. Example: If a person works for the "City of London Police" they could have a verification badge on their social media account. This “**new badge**” would look like this when they post something:

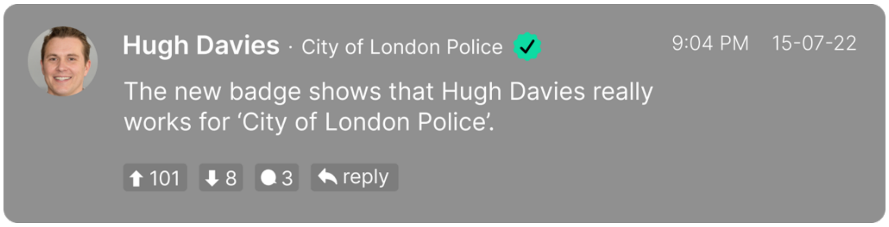

Hence, social media accounts can now have either no verification badge, a classical badge, or a new badge.

Next, we are also experimenting with the possibility to sign social media posts.
These **signatures** can differ per post: one could sign as “verified to work for City of London Police" on one post, and sign as “verified to live in London" for another. Signatures are bound to social media posts, whereas (new) verification badges are bound to the account holders. Here is an example of a signed post:

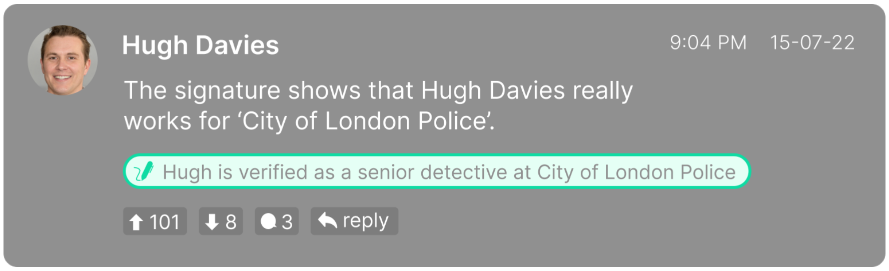

Supplement: S2 File — This text was used to explain to the participants how to interpret the different possible badges included in the second experiment. It was explained that a source could have either no badge, an identity-based badge, a credential-based badge, or signed their message using a credential-based signature. The text further explained how to interpret the badges and signature, namely as verified identity and verified credential respectively. Note that, again, this figure is for illustrative purposes only, as the profile photo is similar but not identical to the one used in the experiment. While the original photo was obtained from Unsplash, this illustrative profile picture was AI-generated through https://thispersondoesnotexist.com. (PDF) [file pone.0302323.s005.pdf]
