# Supplementary material for: Virtual lab coats: The effects of verified source information on social media post credibility
Source: PLoS One. 2024 May 29;19(5):e0302323. doi: 10.1371/journal.pone.0302323 (PMC11135712; doi:10.1371/journal.pone.0302323)
Supplement: Attachment — Submitted filename: Reviewer response letter.pdf [file pone.0302323.s006.pdf]

— PLOS ONE —
Review Response Letter

Virtual Lab Coats paper

October 2023

From the authors

Thank you for your insightful observations and elaborate as well as helpful suggestions to our manuscript. Below, we reply to your remarks individually. The replies include text snippets from the revised manuscript when relevant. We hope that these adjustments address your concerns, improve the manuscript, and contribute to a more comprehensive understanding of our research overall. Your thoughtful feedback is invaluable to us.

Our sincere thanks for taking the time to engage with our work.

Sincerely,
Jorrit Geels, Paul Graßl, Hanna Schraffenberger, Martin Tanis, and Mariska Kleemans

Reviewer #1

Remark 1

The authors framed that the paper's main goal was to empower users toward combating misinformation, and to do this they examined the changes in sharing behaviour before and after informing participants on how to interpret source verification cues. Thus, the study rationale was built on the assumption that sharing is related to judgements of accuracy. The recent studies, however, showed that sharing and truth judgement can be independent/unrelated (Pennycook & Rand, 2021 `https://doi.org/10.1016/j.tics.2021.02.007`). Given this evidence, the rationale behind indexing sharing behavior as a metric for measuring susceptibility to misinformation lacks support from the literature. Also, all the stimuli used in the study were fictional, without proper manipulation of ground truth for posts (e.g., real vs fake). This aspect of the design causes any conclusions made regarding misinformation (or about accuracy of sharing intentions–as the authors wrote in the abstract) from the paper to be erroneous/illogical given there were no ground truth.

Thank you for your detailed feedback and thoughtful considerations, including the very useful academic literature. We appreciate the points you raised.

Regarding the first concern, we recognise that our manuscript has caused some confusion here. Our study was actually built on the rationale that credibility judgements and sharing intentions were not necessarily related. We agree that, if an individual indeed does not find (mis-)information credible but spreads it to others, it is still problematic. We therefore measured and analysed the effects of verified source information on credibility perceptions and sharing intentions separately. We appreciate your observation that this theoretical and practical distinction should have been more prominently emphasised in the manuscript. To address this, we have included this additional paragraph in our related work section to highlight this distinction, starting at line 160:

> Furthermore, many studies have investigated sharing behaviour of users in the context of misinformation (see, e.g., [citations, including the suggested source]). An important notion in this domain is that credibility and sharing judgements have recently been found to be two separate, not necessarily related decisions (see, e.g., [citations, including the suggested source]). For example, users might share information they know to be inaccurate but would be 'interesting if it were true' [citation]. Moreover, misinformation is often shared without sharers being aware of its inaccuracy [citations, including the suggested source]. Hence, credibility judgements and sharing intentions should be considered independently.

Regarding your second concern, we appreciate your insight into the potential value of including ground truth posts in our study. Indeed, we used no ground truth post. We agree that this would have been a valuable addition to the study in hindsight. Our original reasoning was centred on improving the accuracy of the source heuristic in the context of social media, recognising that the accuracy of information is often subjective and dependent on users' perceptions. We aimed to vary the accuracy of the source cue heuristic, rather than the accuracy of the information itself. In hindsight, we acknowledge the potential value of manipulating both information accuracy and theoretical source cue accuracy. We have included this as a valuable suggestion for future work in our discussion (lines 864-880):

> This paper varied the presentation of (verified) source information and its effects on user behaviour, and the relevance of these cues to the message content (i.e. relevant or irrelevant expertise). However, another interesting experimental variation would be to vary information accuracy within the experiments. Using such a ground-truth is common practice in misinformation research. We purposely left this variation out to emphasise that the 'truth' is often emergent and subjective, but future work could certainly experiment with more objective truths or falsehoods.

We hope that these adjustments are an adequate response to your valuable remarks.

Remark 2

In the study, participants were asked to report on their likelihood of sharing and before making any credibility judgements, and the authors reasoned that this was done for preventing any bias (from thinking about credibility) when reporting sharing decision. I am not sure this really helped because the participants were already primed about credibility after viewing questions for the first post. I think the proper experimental technique to deal with this problem is to counterbalance presentation order of questions between (or maybe even within) participants.

Thank you for your thoughtful consideration and feedback on our study methodology. Indeed, because of the two-part structure of the experiment, the participant was already primed to scrutinise the credibility of the social media post in the second part. This noise is inherent to the study design. Amongst other reasons, we therefore decided not to tie binding conclusions to the results from the second half, i.e., they were the result of an exploratory analysis.

Next, we acknowledge your concern about the order of presentation and the potential influence of priming on participants' responses. We view this as a balancing act. Initially, we chose not to randomise the order of measurements, based on the arguments presented in the paper (e.g., sharing intentions could be primed by credibility judgements).

Upon reflection, we agree that counterbalancing would have enhanced the robustness of our experimental design as it is a solid technique to mitigate presentation order effects. We will therefore more carefully consider the implementation of counterbalancing in future studies to address this concern. Hence, we mentioned this as an additional limitation to our research (starting on line 891):

> [...] the experimental survey presented the sharing and credibility items all in the same fixed order. Though this order was fixed to circumvent specific priming effects (sharing intentions could be primed by credibility judgements), other priming effects may have occurred (e.g., sharing intentions priming credibility judgements for internal consistency). Therefore, the results of our study would be more robust if the survey included a randomised presentation order of these items instead.

Remark 3

Was there a measure on the medical knowledge level of participants (e.g., some may medical expertise)? This may have influenced some of the results from the study.

Thank you for this thoughtful remark. Regrettably, we did not incorporate such a measurement of participants' medical knowledge or other forms of prior knowledge in our research design. While we acknowledge that individual variations in prior knowledge could potentially influence certain study outcomes, we believed the impact is likely minimal. Medical knowledge of the level required for potential influence on our results was assumed to be rare. Moreover, we assumed any distribution across conditions to be relatively even. Therefore, we assert that this limitation does not greatly compromise the integrity of our findings. Yet, given that this reasoning is fully based on assumptions, we briefly included this reasoning in the limitations section of the manuscript (starting line 858):

> [...] some participants might have had prior knowledge about the social media posts contents. Though prior knowledge was assumed to be rare and distributed equally among conditions, it might still have affected their credibility judgements (e.g., [citation]). Hence, future research could investigate how verified source information affects user behaviour for users from a wider variety of [...] educational backgrounds.

Remark 4

What was the reason for not including first and second round time point as a factor in the statistical model, but conducting two different statistical models to test effects at these time points instead? This practice may have inflated Type-I errors in results.

Thanks for this valuable critical remark regarding our analysis. We have corrected this mistake and reconducted our analyses. Our analysis with your suggested improvements yielded largely similar results to the initial analysis. Interestingly, the revised analysis revealed that the intuitive viewing of the medical credential badge also increased message credibility for the household context, whereas this was inconclusive in our previous analysis. We have altered our methods section to indicate that we only used one model, edited our results tables to include the model parameters from the improved model, and updated the manuscript to reflect the new results.

Reviewer #2

Major remarks

Remark 1

The introduction and literature review is extensive and much related work is discussed. However, as the manuscript is quite information dense and both the independent variables (types of source verification) and the dependent variables source and message credibility in the study are conceptually quite close to each other, it would help the reader if this section was very clearly structured. In the current setup, the introduction consists of many practical and scientific reasons to conduct this study, but they are not presented in a very clear order. Therefore it took me quite some time to understand what problem the manuscript was exactly addressing and especially what the difference between the different badges was. A more traditional structure of an introduction, in which first the social developments (or problems) are described that the paper aims to solve, followed by the aim and a structured theory section including definitions of key concepts, would prevent the reader (or at least: me) from getting lost. Perhaps some restructuring of this section can also ensure that the background can be shortened a little bit, because I think there was some repetition here and there. On a related note: For me it would help if the practical problem that arises on Twitter (or X nowadays) was first clearly outlined; not only the problem associated with misinformation (which is currently discussed) but also the problem that the currently applied methods of source verification say nothing about source credibility, or message credibility. That point is made later, but since the conflation of source verification and message credibility is quite central to the manuscript, it might be useful to mention this in the beginning.

Thanks for providing valuable suggestions on how to improve the introduction section. In response, we have made large revisions to the introduction and related work sections. Our goal was to enhance clarity by avoiding repetition, emphasising the societal relevance of our research, and reorganising the content to follow the more traditional structure you kindly suggested. For example, we have emphasised the problematic nature of conflating source verification and source credibility, by addressing this concern more prominently in the text. Specifically, we rewrote the first paragraphs to immediately focus on 'verified source information' rather than starting with the general context of 'misinformation' to guide the reader better through the text (starting line 1):

> In our digital- & information-society, misinformation has become a fundamental problem. A recent example includes a wave of misinformation in November 2022, right after Twitter (now 'X') announced it paid subscription [citation]. The new policy included that every user could pay $8 to get a blue verification badge, implying their identity was verified. However, users abused this badge to imply a verified identity with the goal of impersonating companies and spread misinformation. Many readers believed the accounts belonged to the impersonated companies and accepted their misinformation posts as true. Consequently, the stocks of the impersonated companies dropped [citation].
>
> Events like these illustrate the relevance of reliable source information when users look for credible information online. Source information is typically provided by verifying the *identity* of account holders. For company accounts, certainty over their identity is often sufficient to prevent impersonation and misinformation. Yet, for (unknown) people, certainty over their *identity* provides little extra information on the credibility of their information. It can therefore be dangerous if users conflate verified source identity and message credibility.
>
> For more accurate content evaluation, certainty over their *credentials* (e.g., employer, role) can be desired instead [citation], as they could signal expertise. For example, if an unknown person makes a medical claim and one were to doubt its credibility, it is much more valuable to know their medical credentials rather than their identity. Therefore, this paper compares the behavioural effects of source *identity verification* and the novel approach of source *credential verification* to reduce the impact of misinformation in social media.

Your constructive and elaborate feedback have been instrumental in refining our manuscript. We trust that these adjustments address your concerns adequately, and contribute to a more seamless and less effortful reading experience.

Remark 2

The background extensively discusses source credibility and message credibility as dependent variables, but sharing intentions are less discussed. The importance of this variable is briefly mentioned from a practical perspective, but less from a theoretical/literature angle. Later in the manuscript, the authors indicate that credibility evaluations and sharing intentions do not necessarily have to be related and that the latter are also influenced by other factors. Nevertheless, these variables are often examined together in studies and it might therefore be good to pay more attention to the (theoretical) background of this variable, even if the variables are less related than expected.

Thanks for this accurate observation regarding our coverage of all measured concepts in our related work section. We agree that the section needed a more in-depth exploration of the theoretical background of sharing intentions. Therefore, we have revisited this section and expanded it to better highlight this variable. Specifically, we added these paragraphs (starting on line 160):

> Furthermore, many studies have investigated sharing behaviour of users in the context of misinformation (see, e.g., [citations]). An important notion in this domain is that credibility and sharing judgements have recently been found to be two separate, not necessarily related decisions (see, e.g., [citations]). For example, users might share information they know to be inaccurate but would be 'interesting if it were true' [citation]. Moreover, misinformation is often shared without sharers being aware of its inaccuracy [citations]. Hence, credibility judgements and sharing intentions should be considered independently.[1]
>
> The relationship between source credibility and sharing has been explored in a similar research by Kim & Dennis [citation]. In their experiment, some posts included source credibility ratings in the form of, e.g., star ratings. They found that when including such credibility ratings, users became more critical of all incoming information and decreased in sharing behaviour. These results illustrate how the design of social media posts can affect both credibility and sharing behaviours. The authors call for investigations on how author information influences credibility and sharing behaviour. The present paper investigates the effects of two types of (verified) author information: identity verification, and credential verification. Related work on either approach is discussed below.

We hope these added paragraphs are in line with your expectations.

Remarks 3 & 5

Remark 3: On page 6 (line 222) the authors state that they use Bayesian analyses to obtain more certainty about the relationship between source credibility cues and the measures. What is meant by "more certainty"? And compared to what exactly?

Remark 5: Hypothesis 1 a/b/c assumes no effect of the independent variable on the dependent variable. As I am not a statistician I cannot properly judge whether this is a problem or not. I appreciate the extensive analysis section. Yet it is still difficult to understand for readers with limited knowledge of Bayesian models. In particular, I would like to read here how this type of statistic is suitable for testing hypotheses without an effect.

Thanks for your honest remarks. We acknowledge that the use of Bayesian models requires clearer motivation, especially as they are still not that common. Hence, we changed the manuscript to delve a little deeper in why Bayesian models are a suitable method for testing hypotheses without an effect. Specifically, we mention this in our revised manuscript in the background section (beginning on line 211):

> [...] the effects of identity verification on source and message credibility as well as sharing behaviour should be explored more in-depth using methods that can detect whether an effect is present, e.g., Bayesian models (e.g., [citation]). Namely, in contrast to classical frequentist statistical methods, Bayesian models can both reject as well as *accept* null hypotheses.

Next, the revised manuscript emphasises this again when introducing study 1 (line 259):

> As noted above, previous work in this area used frequentist statistical methods, which, in contrast to Bayesian methods, cannot yield conclusions on the presence or absence of an effect (e.g., [citation]). Hence, the data were analysed with Bayesian models to determine the presence or absence of the relationship between source credibility cues and the aforementioned measures. Another important reason to conduct a Bayesian analysis instead of a frequentist analysis, is because Bayesian models often align more closely with how humans conceptualise and interpret parameter estimates [citation].

Remark 4

The authors state that the hypotheses of study 1 are based on Vaidya's study. Because this study has such an influence on this paper, it would be helpful to the reader to get a little more background about this study. For example, what do the authors provide as the reason that there is no difference in effect on the dependent variable between the messages with and without a badge?

Thanks for your suggestion. We have revised the manuscript to incorporate more details about Vaidya et al.'s study, including the motivation behind their conclusions. Specifically, the revised manuscript now reads (line 191):

> Some scholars did not detect a relationship between account verification and message credibility [citations, including Vaidya et al.]. This suggests users may simply not conflate source authenticity and message credibility. For example, Vaidya et al. state "users generally understand the meaning of verified accounts" [citation of Vaidya et al.] (p11).

Moreover, when introducing our hypotheses, we more explicitly refer to how our hypotheses differ from Vaidya et al.'s findings with respect to our conceptualisation. This passage now reads the following (line 255):

> To enable easier comparison of results, we adopted the stimuli and hypotheses 1a/b/c based on the work by Vaidya et al. [citation]. Next, study 1 used a similar methodology, thus focusing on Twitter. Yet, the study differs in the conceptualisation of credibility. Namely, we separately measured source credibility, message credibility, and sharing intentions.

[1] It is worth noting that the first paragraph includes the Pennycook & Rand paper kindly suggested by Reviewer #1.

We believe that this addition will contribute to a more comprehensive understanding of the foundation upon which our hypotheses in Study 1 are based.

Remark 6

A power analysis was performed to determine the number of subjects. Is this also suitable for Bayesian analyses?

Indeed, our power analysis was applicable to frequentist statistic approaches. However, although Bayesian statistic analysis can already be reliably performed using a small number of samples, it does benefit from using more samples. Hence, to determine and motivate an 'ethical' / 'right' number of participants, we opted for using the participants applicable for a frequentist approach. Moreover, this enables others to conduct frequentist analyses on our data without the limitation of power, contributing to open science. We briefly included this reasoning in the revised manuscript for transparency (beginning on line 333):

> [...] we recruited a total of $N = 525$ participants, based on a G*power analysis. Note that this analysis is typically only used for frequentist statistic analyses, as opposed to Bayesian analyses. Still, the G*power analysis was purposely used to indicate an 'ethical' sample size and to enable other researchers to re-use our data for frequentist analyses.

Remark 7

The results for both studies consist of two parts; outcomes for the intuitive response and for the informed response. Ultimately, the results also show that there are considerable differences between them. This shows that the 'explanation about the interpretation of the badges' is crucial, especially because the authors ultimately believe that it does more harm than good. That is why, as a reader, I would like to see the exact explanation that the participants received, in the text, or as a table or figure. This would be informative. In addition, it might be good if the authors delved a little deeper into the theoretical implications of this finding in the discussion. Now this mainly happens on a practical level - for example by finding that simple media literacy interventions may not work sufficiently.

Thank you for raising these two thoughtful remarks on the exploratory information intervention included in both studies.

In response to your first point, we have included the detailed explanation of badge interpretation as supplemental material. While we understand the importance of transparency, we opted for a supplemental material rather than in-text inclusion to maintain the focus and scope of the main text. The impact of information is approached as an exploratory measure, and we aimed to strike a balance in providing transparency without deviating too much from the main narrative.

Regarding your second point, we appreciate the suggestion to delve deeper into theoretical implications. However, we believe that connecting theoretical implications to our results of the informed viewing would not align with the exploratory nature of these findings. Hence, instead of discussing theoretical implications, we now call for future work on such theoretical investigations of how information affects credibility judgements and sharing intentions. This is discussed in the following paragraph, starting on line 836:

> As most reasoning errors occurred after informing participants about the verified source information cues and what they mean, purely explaining how to interpret them seems insufficient to overcome the prevalence of these reasoning errors. In other words, this simplest form of a (news) media literacy intervention did not have the anticipated effect. This calls into question to what extent such low profile interventions are useful in combating misinformation, and subsequently how media literacy interventions should look like to be effective. Past research (e.g., [citations]]) indicates that more sophisticated interventions (e.g., integrating several strategies and/or featuring multiple messages) are promising. However, it is crucial to note that our theoretical suggestions are based on exploratory research, and we refrain from making definitive conclusions. Further investigation is required, particularly when it comes to the existence of negative side-effects of exposure to interventions [citation], as also found in the current study.

We hope that these adjustments address your concerns and contribute to a more comprehensive understanding of our research.

Remark 8

To be honest, I found it hard to interpret the tables. Presenting the dependent variable on the far left column is a bit counter intuitive. But I had the greatest difficulty with the estimate that was presented. What does this entail? Is this a difference score between the average score with badge and without badge? For me it would be much more informative if the mean scores on each dependent variable were presented for the different types of badges (and no badge). The difference scores (or effect sizes) that are now in the table could be included in the text when the authors discuss a specific effect.

Thank you for pointing this out to us, it is very much appreciated. The current tables are indeed rather specific, though standard to Bayesian analyses. Yet, we acknowledge that our analysis is not a mainstream analysis to conduct. Therefore, we included more explanation on how to interpret the tables in the manuscript. For example, the note below table 1 (right below line 460) now reads:

> This table shows the contrasts between the identity badge condition and no badge condition for both credibility measures and sharing likelihood. First, we observed the 95% credible intervals (CrIs) of each estimate and checked whether the CrI fell into the ROPE range. If the full CrI fell into the ROPE range (ranging from -0.1 to 0.1), there is an effect. If the CrI fell entirely outside the range, there is no effect. Present effects are marked with an asterisk (*). If the CrI and ROPE overlapped, we cannot draw a conclusion about the presence or absence of an effect given our data. The estimate and its standard deviation (SD) indicate how the contrasts are distributed.

Remark 9

It might be good to provide some reflection in the discussion section on the choice of testing these effects with false or accurate information provided in the messages. This varied across the two studies if I understood correctly (study 1 false information, study 2 accurate information) but could this potentially have impacted people's evaluation? What is the role of prior knowledge here?

Indeed, study 1 used inaccurate information whereas study 2 used accurate information. Study 1 adopted Tweets including inaccurate information from the Vaidya et al. (2019) paper, as study 1 focused on confirming their findings. However, for study 2 these posts were revised to include accurate information. Namely, we did not want to bias credibility or sharing results with statements participants potentially knew to be false. Also in response to reviewer #1's remark 1, we added this to our discussion section, specifically at lines 864-880:

> This paper varied the presentation of (verified) source information and its effects on user behaviour, and the relevance of these cues to the message content (i.e. relevant or irrelevant expertise). However, another interesting experimental variation would be to vary information accuracy within the experiments. Using such a ground-truth is common practice in misinformation research. We purposely left this variation out considering that the 'truth' is often emergent and subjective, but future work could certainly experiment with more objective truths or falsehoods.

The social media post contents were thus chosen to be ambiguous in quality to the user. In that case, the user resorts to source cues (see, e.g., Chaiken & Maheswaran, 1994). Indeed, if the quality was not ambiguous to the user, the source cues would have less of an effect. Still, given the topics used, we deemed participants with relevant background knowledge to not influence our results. Also in response to reviewer #1's remark 3, we added the following snippet to the manuscript to elaborate on the role of prior knowledge (starting line 858):

> [...] some participants might have had prior knowledge about the social media posts contents. Though prior knowledge was assumed to be rare and distributed equally among conditions, it might still have affected their credibility judgements (e.g., [citation]). Hence, future research could investigate how verified source information affects user behaviour for users from a wider variety of cultural and educational backgrounds.

Remark 10

The authors indicated that the data of the studies are publicly available, but for me it was unclear how to access the data. This could be a problem with the system, however, it means that I could not review the data.

Thanks for noticing. We seemed to be confused with the system. In response to your remark, we now made the data public on our open science framework pages linked in the manuscript. These are `https://osf.io/cyh8e/` *(study 1) and* `https://osf.io/y2uew/` *(study 2).*

Minor remarks

Remark 1

The authors state that a generic name and profile photo have been chosen for the Tweets, from the appendix I deduce that the sender is a woman. Was there a consideration behind this?

Thanks for your remark. To clarify, the fictional sender was indeed a female. This was chosen somewhat arbitrarily. Still, while on the one hand we acknowledge that gender of a source can (unfortunately) affect their credibility, on the other hand the stimuli source gender was kept constant over the studies and conditions. Hence, we can ascribe the observed effects to the other source cues. Varying the gender of the source was considered outside of scope for our research.

Remark 2

I think the measures section could be a little more structured. Now the concepts are hidden in the long paragraph. A paragraph per measure is clearer.

Thanks for this remark! In response, we have revised the measures section to provide a more structured presentation. Each measure now has its dedicated paragraph (see pages 9 and 16), aimed at improving readability and clarity of the articulated concepts. We hope that this adjustment aligns with your expectations.

Remark 3

The authors use the media literacy scale of Vraga et al. Is this a validated scale? And can the authors provide some sample items? The description of this measure refers to item 6, but without further representation of the scale, 'item 6' has no meaning for the reader. Better to give the substantive item.

Thanks for your remark. We want to clarify that the media literacy scale employed in our study is indeed validated. Next, to enhance the clarity of our presentation, we now have included some sample items in the revised manuscript (line 378-383):

> Lastly, media literacy skills were assessed using the validated scale from Vraga et al. [citation]. This scale is comprised of six items on a seven-point scale ranging from *Strongly disagree* (1) to *Strongly agree* (7) [citation], $M = 5.51, SD = 0.84$, $\alpha = .78$. Example items are "I have the skills to interpret news messages" and "I'm often confused about the quality of news and information".

We recognise the importance of providing concrete information to enables readers to grasp the used measures. We hope that the inclusion of these sample items will address your concern.

Remark 4

Why was trust in organisations not added as a control variable and twitter use and media literacy included?

When performing the analysis, we carefully considered the potential impact of trust in organisations on our results. However, after a thorough examination, we found that controlling for this variable did not yield any substantive alterations to the outcomes presented in the manuscript. It's important to note that the decision not to include trust in organisations as a control variable was made with deliberate consideration. Unfortunately, validated scales or peer-reviewed statements specifically measuring this variable were not readily available to us during the course of our study. Consequently, we opted to focus on variables with established measures to ensure the robustness and reliability of our findings.

We elaborated on this in our revised manuscript. We now mention the following on this matter (see line 417 and following):

> The model controlled for the participants' Twitter use and media literacy. Trust in medical organisations was excluded for robustness and reliability, i.e., because this factor was not measured using a validated scale. Note that controlling for this variable yielded mostly similar outcomes to the main analysis.

Remark 5

Page 2, line 10: this states that the paper examines 3 ways to counter misinformation on social media, but more precisely: ways to counter the impact/effects of misinformation.

Thanks for the suggestion. We gladly took this comment with us in revision of our introduction, and improved the line as follows (line 19):

> [...] Therefore, this paper compares the behavioural effects of source *identity verification* and the novel approach of source *credential verification* to reduce the impact of misinformation in social media.

Remark 6

Other wording issue: page 18, line 753 states that for the intuitive viewing participant behaviour was unpredictable. I would advise the authors to be more precise here, for example that in this case the source credibility cue did not impact message evaluation.

We made it more explicit in the new manuscript what we mean by 'unpredictable'. To elaborate, we explained that unpredictable means that the evidence does not clearly indicate the presence/absence of an effect. The revised manuscript now reads, from line 809 onwards:

> Before explanation, participant behaviour was unpredictable in a general social media setting. Namely, in most cases, both identity and credential verification methods did not show a clear absence or presence of effects on sharing intentions, and source or message credibility. Our results thus question the omnipresence of verified identity markers on social media platforms, as they do not seem to increase source credibility intuitively.

Remark 7

The term “truth evaluations/assessment” appears in various places in the manuscript. For example on page 2, line 22. The author may consider replacing this with accuracy evaluations.

Thanks for the suggestion. Indeed, our paper focuses on subjective accuracy evaluations rather than ‘objective’ truth evaluations. We changed the phrasing of truth evaluations or truth assessment in the manuscript to accuracy evaluations.

Remark 8

Page 4, line 112; in should be an; Page 5, line 161; represent represent (redundant word). Same applies to page 13, line 520.

We apologise that these mistakes passed all authors. We changed all items in the manuscript.

Remark 9

Page 19, line 792: In my view ’explanation for this finding’ better fits the context than ’explanation for this data’.

Thanks for the suggestion. The revised manuscript now reads (line 849):

> A likely explanation for this finding is that [...]
